# Antarctic Salt-Cones: An Oasis of Microbial Life? The Example of Boulder Clay Glacier (Northern Victoria Land)

**DOI:** 10.3390/microorganisms10091753

**Published:** 2022-08-30

**Authors:** Maurizio Azzaro, Maria Papale, Carmen Rizzo, Emanuele Forte, Davide Lenaz, Mauro Guglielmin, Angelina Lo Giudice

**Affiliations:** 1 Institute of Polar Sciences, National Research Council (CNR-ISP), 98122 Messina, Italy; 2 Stazione Zoologica “Anton Dohrn”, Marine Biotechnology Department, Sicily Marine Centre, Villa Pace, 98168 Messina, Italy; 3 Department of Mathematics and Geosciences, Trieste University, 34128 Trieste, Italy; 4 Department of Theoretical and Applied Sciences, Insubria University, 21100 Varese, Italy

**Keywords:** extremophiles, salt cone, mirabilite, thenardite, prokaryotic diversity, supraglacial systems

## Abstract

The evaporation of a localized, highly saline water body of the Boulder Clay debris-covered glacier, in the Northern Victoria Land, probably generated the accumulation of mirabilite (Na_2_SO_4_ × 10H_2_O) and thenardite (Na_2_SO_4_) in a glacier salt-cone. Such an extremely cold and salty environment resembles the conditions on Mars, so it can be considered a terrestrial analog. The study was aimed at gaining a first glimpse at the prokaryotic community associated with Antarctic mirabilite and thenardite minerals and also to find clues about the origin of the salts. For this purpose, samples were analyzed by a next generation approach to investigate the prokaryotic (Bacteria and Archaea) diversity. Phylogenetic analysis allowed the identification of *Bacteroidota*, *Actinobacteriota*, *Firmicutes*, and *Gammaproteobacteria* as the main bacterial lineages, in addition to Archaea in the phylum *Halobacterota*. The genera *Arthrobacter*, *Rhodoglobus*, *Gillisia*, *Marinobacter* and *Psychrobacter* were particularly abundant. Interestingly, several bacterial and archaeal sequences were related to halotolerant and halophilic genera, previously reported in a variety of marine environments and saline habitats, also in Antarctica. The analyzed salt community also included members that are believed to play a major role in the sulfur cycle.

## 1. Introduction

The interest in extreme environments has grown in recent years to understand what the real limits of the survival of species are and to what extent life goes to persist. Hypersaline environments are present on our planet in all continents, including coastal lagoons, salt and soda lakes, briny pools, brine channels in sea-ice and subsurface brine deposits. To be classified as hypersaline, an environment should have high salinity levels (superior to seawater) and could be also saturated.

Antarctica hosts several subsurface hypersaline liquid cryobrines that reach the eutectic point (i.e., the point at which all compounds, including water, pass to the solid state) at temperatures below 0 °C. This makes such Antarctic saline cryoenvironments possible terrestrial analogs of Mars [1]. In fact, geomorphic and geochemical observations suggest the occurrence of liquid water or brines in the subsurface of the red planet [2], possibly representing a protected environment for potential actual or past life. More interestingly, evaporite deposits, probably deriving by eruptions of subsurface fluids (mounds), were discovered on Mars [3,4].

The presence of salt deposits is generally related to warm and arid environmental conditions. However, some salts form by cooling and a concentration mechanism based on cooling and/or freezing. In fact, the freezing process acts similarly to the evaporation in the concentration of saline liquids (called frigid and evaporative concentration, respectively), as water is removed from the solution by the formation of ice, leading to a concentrated residue and the progressive precipitation of saline minerals [5]. The minerals formed under these conditions are known as cryogenic [6,7].

Salt masses, probably formed during glacial advances, have been detected in relation to glaciers [8] or in the ice, and results constituted of mirabilite (Na_2_SO_4_ × 10H_2_O). However, there is not detailed information about their origin and composition. Some salt discharges were observed in the past in Victoria Land, Antarctica, and were attributed to deposits coming from the Taylor Glacier. The salt composition was detected as a mixture of halite (NaCl), aragonite (CaCO_3_) and other salts, and was compared to the composition of the water bodies occurring in the area. However, the authors established the difficulty in identifying the origin of salt deposits by only analyzing and comparing their composition and suggested the improvement of physical setting and geomorphic history for a correct interpretation [9]. Bowser et al. [10] according to bulk composition and isotopic data, hypothesized a seawater source for the sulfate and a glacial ice source for the water of crystallization of mirabilite. The mirabilite deposits formed by freeze concentration in ponds fed largely by glacial meltwater. Possibly, a mechanism of bacterial sulfate reduction and subsequent reoxidation within the meltwater ponds can be imagined. Later on, Liu et al. [7] confirmed the mirabilite-mounds are deduced to have formed via a freezing/sublimation process that occurred in glacial or subglacial bodies of water supplied by glacial tills containing microbially oxidized sulfate ions

These kinds of habitats are considered extreme due to the stress imposed on cells through the strong salinity gradient between intra- and extracellular environments. Microbial communities, at the basis of the functioning of all ecosystems, have been widely proven to have great adaptability and resistance capacities even in the presence of very adverse conditions. However, the adverse conditions of such environments affect the microbial community diversity, by favoring the only few taxonomic groups that can survive adapting to such peculiar conditions. Among extremophilic microorganisms, halophiles are known to be able to tolerate salty habitats thanks to special mechanisms of adaptation. Indeed, they accumulate or produce nontoxic solutes to maintain the internal environment isosmotic with the external environment and use special proteins to avoid denaturation at high salt concentration. Halophiles dominate the microbial communities and despite in many cases they are mainly represented by several groups of archaeal microorganisms, in some hypersaline environments the predominance is given by Bacteria. They are also of special interest for astrobiological perspectives, due to the strong similarities of hypersaline environments with Martian surfaces, i.e., high salt concentration and strong UV levels.

The present research was aimed at studying salt efflorescences from the Boulder Clay Glacier (Antarctica) debris-cone from the perspectives of microbial diversity, as a hot spot of microbial life, in extreme environmental conditions, comparable to extraterrestrial life. Mineralogical analyses were, moreover, performed to investigate the minerals occurring in the salt efflorescences.

## 2. Materials and Methods

### 2.1. Sampling Area

**Figure 1:**
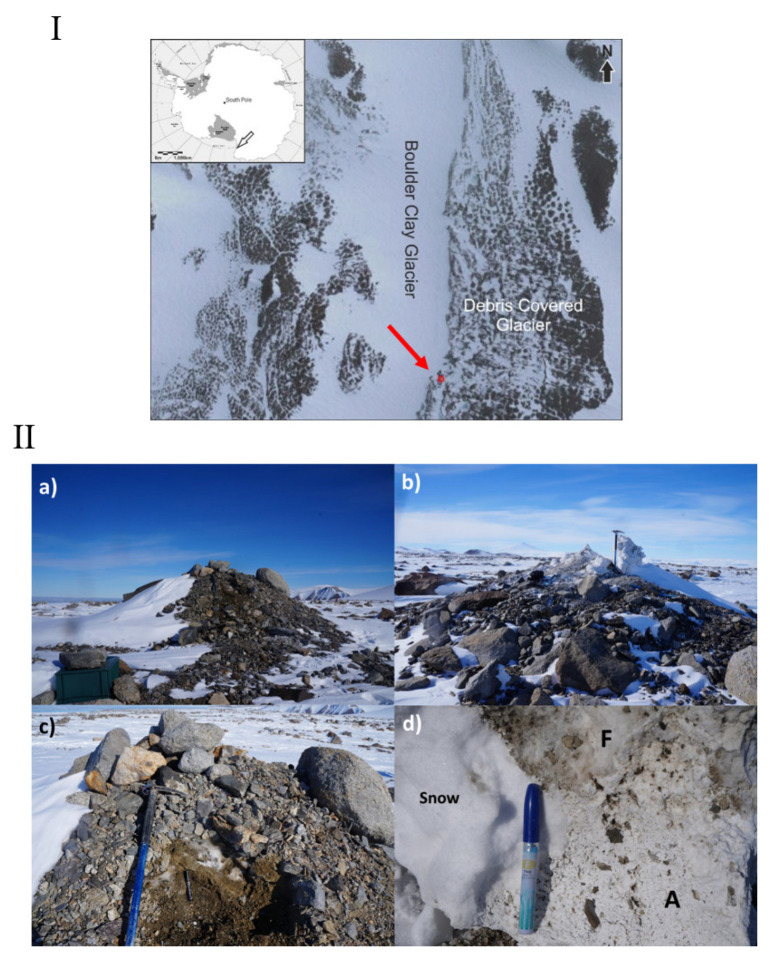
(**I**) Location map. The sampled salt cone is indicated by a red point. Satellite image taken in November 2014 (courtesy Maxar technologies). (**II**) Salt cone photographs. Cone view from the East (**a**) and West (**b**) side of the Boulder Clay glacier. Details of the salt cone (**c**), and fresh (F) and altered (A) crystals (**d**).

The Boulder Clay (BC) area is about 6 km south of the Italian station “Mario Zucchelli” on a very gentle slope (5°) with southeastern exposure. The area is characterized by a glacier in the inner part and a debris covered glacier mantled by ablatio-sublimation Late Glacial till, the “Younger Drift” cited by Orombelli et al. [11]. The till is in permafrost condition with a very thin active layer (ranging between 23 and 92 cm increasing since 90ths) [12,13]. In particular, the sampling site is located along the margin of the BC debris-covered glacier adjacent to the homonymous permafrost monitoring station of Boulder Clay (Figure 1I). Here, the mean permafrost temperature at the permafrost table depth (30 cm) ranged between −15 and −17 °C [14].

### 2.2. Sample Collection

The cone was about 3.5 m high (Figure 1II(a)). Its stratigraphy, from the surface to the bottom, encompasses moraine materials (5–15 cm thick) with some blocks and boulders, altered and fresh semi-transparent crystals (Figure 1II(c)), compact ice with vertical bubbles and sediments. Close to the surface the materials were altered into a white powder, while in contact with ice the crystals seemed to be stable.

Such type of salt is also common on the pebbles and on the blocks embedded in finer material in the surrounding areas, up to the average height of the wind drift snow. These salty encrustations are due mainly to the thermal effect of the pebbles and blocks. In fact, sun radiation increases temperature more significantly and rapidly, inducing the snow melting and water evaporation from the surrounding finer material. The sampled salt cone was already studied by Chinn [15] who suggested the origin of the sediment as the product of a localized, highly saline water body, such as a kettle or ice-marginal lake.

**Figure 2:**
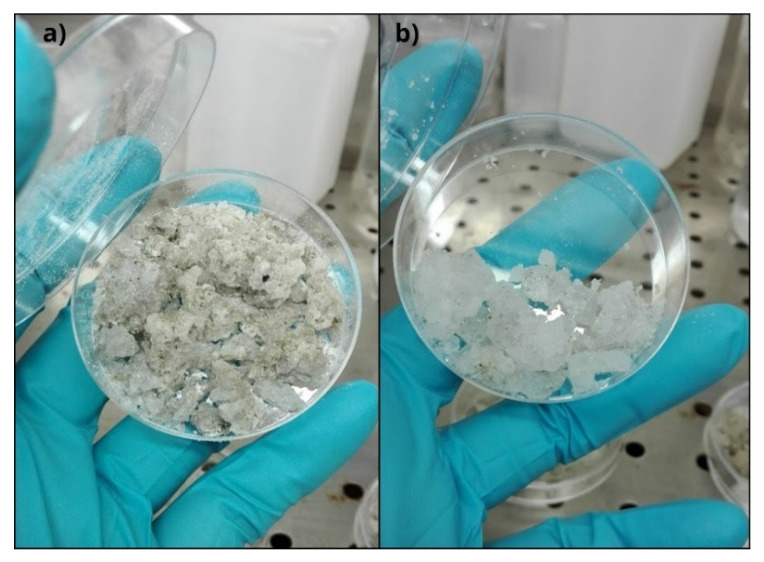
Salt samples analyzed in this study. (**a**) Dark salt (DS); (**b**) White salt (WS).

The collection of salt samples was performed after removing the external layers above the salt cone. Samples for DNA extraction were aseptically collected from both fresh crystals, from about 5 cm below the cone surface, and in the proximity of sediment, resulting in two colors, i.e., white (WS) and dark (DS) salt, respectively (Figure 2). The sampling was conducted taking care to collect the two types of salt separately. Fresh semi-transparent crystals (from about 5 cm below the cone surface) were collected for mineralogic analyses. All samples were stored at −20 °C until analysis.

### 2.3. Mineralogical Analysis

Powdered samples were analyzed via X-ray Powder Diffraction using a STOE D 500 X-ray diffractometer (Siemens, Berlin, Germany) at room temperature. The CuKα radiation was used through a flat graphite crystal monochromator. The current used was 20 mA and the voltage was set at 40 kV. The 2θ scanning angle ranged from 2 to 60°, with 0.01° steps and a counting time of 2 s/step.

### 2.4. Prokaryotic Community Composition

#### 2.4.1. DNA Extraction and NGS Sequencing

The prokaryotic community composition was evaluated in salt samples. Total DNA was extracted directly from the salt samples in triplicate, using the Power Soil DNA extraction kit (MoBio Laboratories, Carlsbad, CA, USA) according to the manufacturer’s instructions. DNA concentrations and purity were quantified by using a NanoDrop ND-1000 UV-vis spectrophotometer (NanoDrop Technologies, Wilmington, DE, USA). Bacterial 16S rDNA region V3-V4 was amplified using universal primers 341F 5′-CCTACGGGNGGCWGCAG-3′ and 805 R 5′-GGACTACHVGGGTATCTAATCC-3′, and the adapter sequences, added to the locus-specific sequencing were 16S F 5′-TCGTCGGCAGCGTCAGATGTGTATAAGAGACAG-3′ 16S R 5′-GTCTCGTGGGCTCGGAGATGTGTATAAGAGACA-3′ [16]. Sequencing was performed using the Illumina MiSeq platforms, following the standard protocols of the company IGA Technology Services Srl (Udine, Italy).

#### 2.4.2. Bioinformatic Analysis

FastQC was used to check the quality of raw sequences [17]. Sequences were pre-processed, quality filtered, trimmed, de-noised, merged, modeled, and analyzed by R package DADA2 [18] to infer amplicon sequence variants (ASVs), i.e., biologically relevant variants rather than an arbitrarily clustered group of similar sequences. During the analysis, filters for reducing replicate, length, and chimera errors were also applied. Bacterial taxonomy annotation was performed using Silva database formatted for DADA2, offering an updated framework for annotating microbial taxonomy (silva_nr99_v138.1_wSpecies_train_set.fa.gz and silva_species_assignment_v138.1.fa.gz). A taxa filter was used for the decontamination from eukaryotic, chloroplast, and mitochondrial sequences. Finally, a manual inspection was done, and sequences with ≤0.1% abundance were not considered.

#### 2.4.3. Statistical Analyses

The occurrence of significant differences among abundance values in salt samples was tested by One-way ANOVA test and post hoc analysis (Tukey test), by considering results statistically significant when *p* < 0.05. Results of relative abundances were transformed and processed by calculating the Bray—Curtis similarity. The similarity matrix was used to compute the non-metric multidimensional analysis (nMDS).

## 3. Results

### 3.1. Mineralogical Analyses

**Figure 3:**
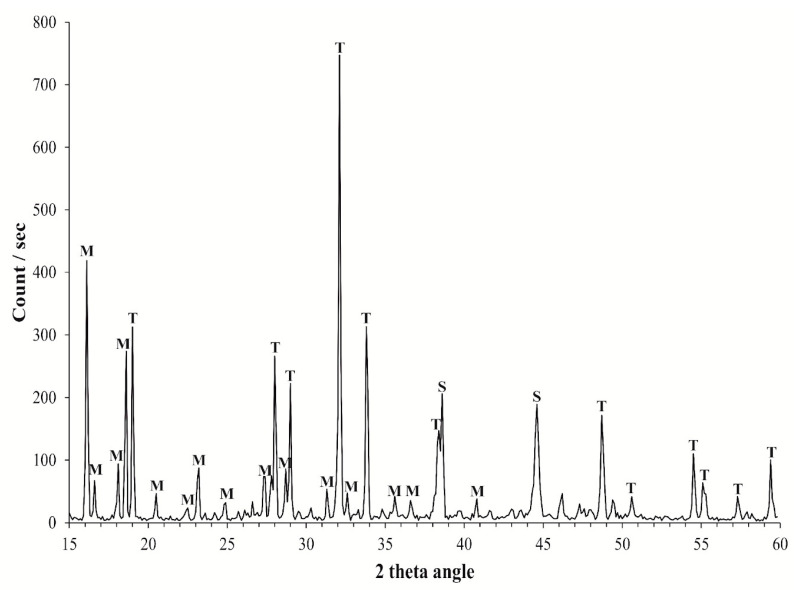
X-ray powder diffractograms of salt crystals. M: mirabilite; T: thenardite; S: sample holder.

The XRD pattern of the samples shows the presence of two minerals, namely thenardite (Na_2_SO_4_) and mirabilite (Na_2_SO_4_ × 10H_2_O) (Figure 3). Within the samples the ratio mirabilite/thenardite was different. In the figure below it is represented by the sample where there was the higher content of mirabilite.

### 3.2. Prokaryotic Community Composition

**Figure 4:**
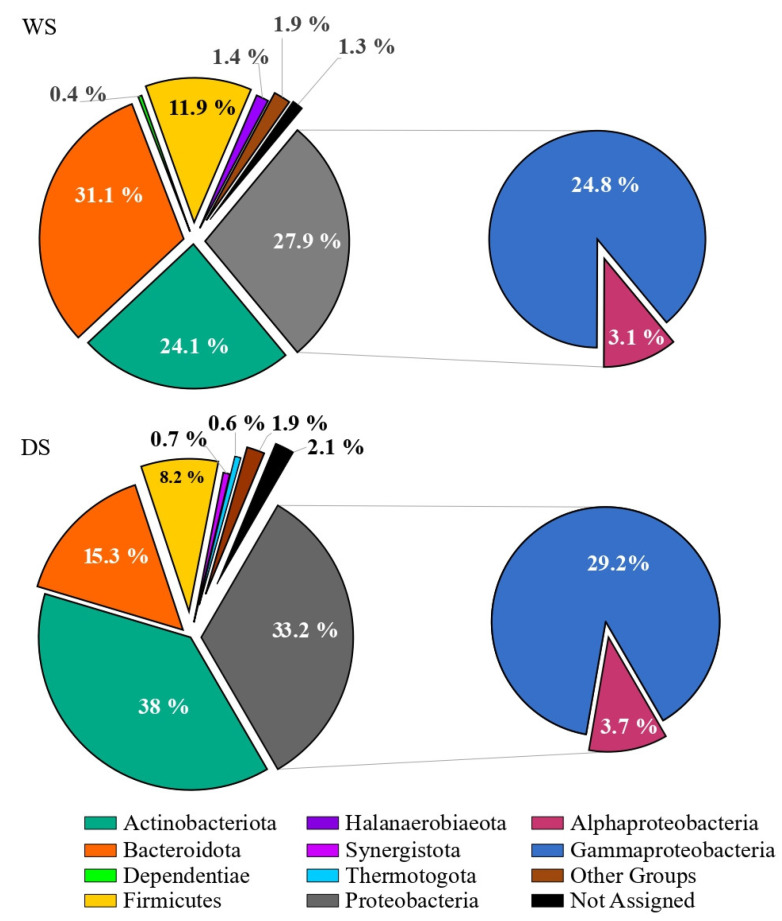
Comparison of phylum distribution overall observed in white and dark salt samples. *WS*, white salt; *DS*, dark salt.

Main data on total sequence reads, quality trimming, ASV information and diversity indices obtained for salt samples are summarized in the Supplementary Table S1 and Figure S1. The overall taxonomic composition of prokaryotic communities in salt samples is shown in Figure 4. In WS, ASVs were mainly related to *Bacteroidota* (31.1 % of total sequences), followed by *Proteobacteria*, *Actinobacteriota* and *Firmicutes* (27.9, 24.1 and 11.9%, respectively). *Proteobacteria* were represented by *Alpha*- and *Gammaproteobacteria* with a respective percentage of 3.1 and 24.8%. The WS also hosted members of *Halanaerobiaeota* and *Dependentiae*, even if to a minor extent (1.4 and 0.4%, respectively). In DS, ASVs were mainly related to *Actinobacteriota* and *Proteobacteria* (38 and 33.2 % of total sequences, respectively), followed by *Bacteroidota* and *Firmicutes* (15.3 and 8.2%, respectively). *Proteobacteria* were represented by *Alpha*- and *Gammaproteobacteria* (3.7 and 29.2%, respectively). Members of *Synergistota* and *Thermotogota* (absent in WS) occurred in DS samples (0.7 and 0.6%, respectively). In both samples, minor groups (<1%) accounted for 1.9% of total sequences, while the 1.3 and 2.1% (in WS and DS, respectively) were not assigned at phylum level.

**Figure 5:**
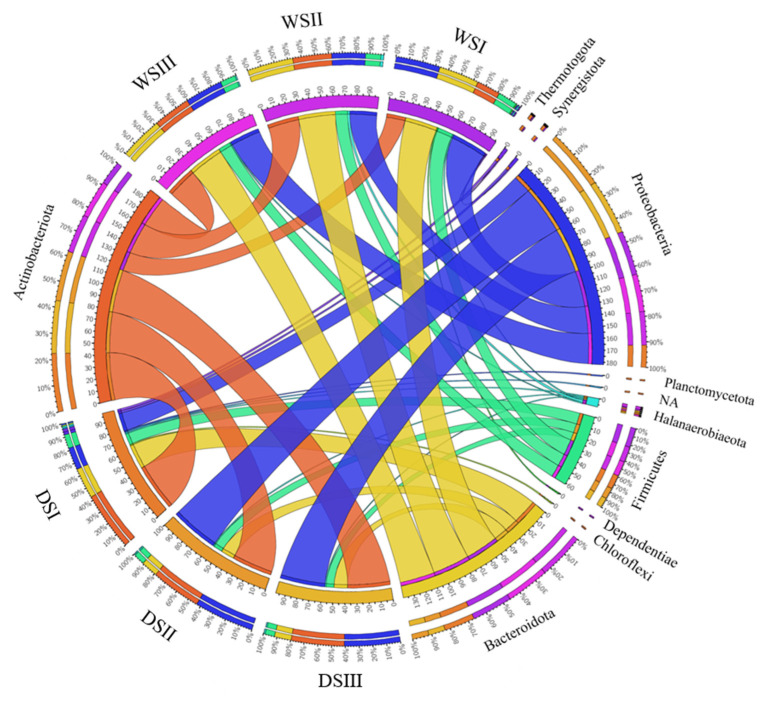
Circos diagrams showing the connection among salt samples and main prokaryotic phyla. The ribbons show ribbon contribution for each segment. Only taxonomic groups with relative abundance >1% are represented here. *WS*, white salt; *DS*, dark salt.

WS samples appeared to be homogeneous and comparable, except for *Actinobacteriota* having a lower relative abundance in WSI (Figure 4 and Figure 5).

Differently, among dark salt samples, DSI was quite different in taxonomic composition compared to DSII and DSIII, due to the presence of *Thermotogota* and *Synergistota* and a minor occurrence of *Proteobacteria* (Figure 5).

**Figure 6:**
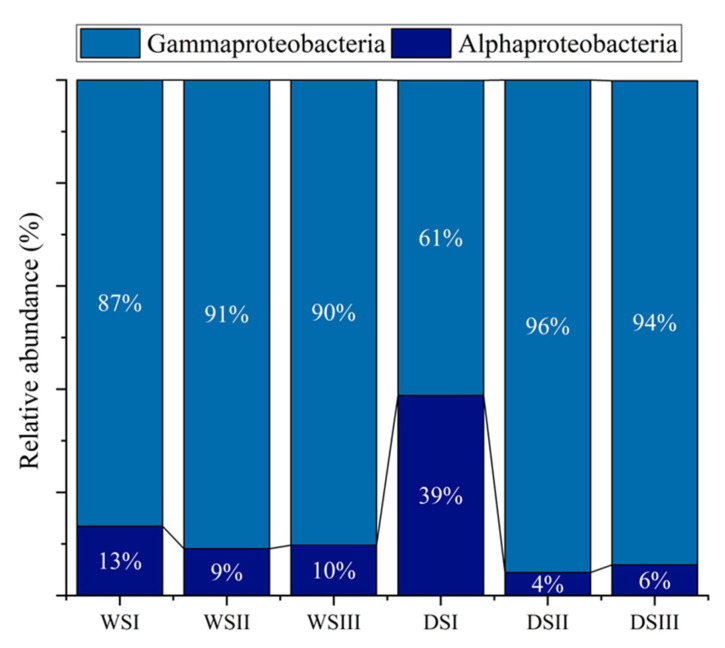
Distribution of *Proteobacteria* taxonomic groups detected in salt samples. *WS*, white salt; *DS*, dark salt.

*Proteobacteria* reads were predominantly affiliated to *Gammaproteobacteria* in all samples, with relative percentages ranging from 61 to 96%, followed by *Alphaproteobacteria* with relative percentages between 4 and 39% (the highest in DSI sample) (Figure 6).

**Figure 7:**
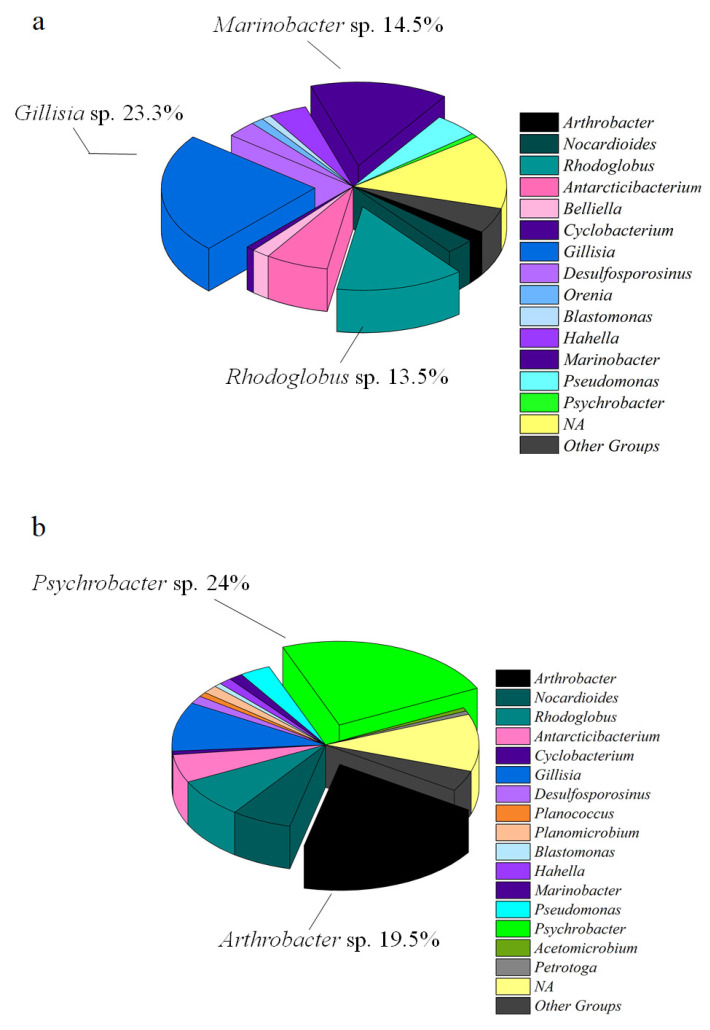
Taxonomic composition at genus level for white (**a**) and dark (**b**) salt samples.

At genus level, with the exception of *Belliella*, only few genera occurred at >1% in at least one salt sample and, generally, in both samples. *Arthrobacter* and *Rhodoglobus* (among *Actinobacteriota*), *Gillisia* (among *Bacteroidota*), *Marinobacter* and *Psychrobacter* (among *Proteobacteria*) were particularly abundant (range 12.4–21.9%). Overall, considering globally the white salt and dark salt samples, the predominance at genus level of *Gillisia*, *Marinobacter* and *Rhodoglobus* was detected in WS samples (expressed as the average abundance among the three WS samples; Figure 7a), whereas the predominance at genus level of *Psychrobacter* and *Arthrobacter* was detected in DS samples (expressed as the average abundance among the three DS samples; Figure 7b).

In WS samples, *Proteobacteria* were mainly represented by *Marinobacter* (10, 15 and 14% in WSI, WSII and WSIII, respectively), *Pseudomonas* (5, 3 and 4% in WSI, WSII and WSIII, respectively) and *Hahella* (6, 1 and 3% in WSI, WSII and WSIII, respectively) (Table 1). *Bacteroidota* were mainly represented by *Gillisia* (25, 18 and 21% in WSI, WSII and WSIII, respectively), and *Firmicutes* by *Desulfosporosinus* (4% in WSI, and 3% in both WSII and WSIII). Differences occurred among WS samples in the *Actinobacteriota* occurrence. *Rhodoglobus* member predominance was recorded in WSII and WSIII (19 and 14%, respectively), while in WSI most ASVs related to this taxonomic group were not assigned.

In DS samples, among *Actinobacterota* the genus *Arthrobacter* was predominant in DSII and DSIII (28 and 25%, respectively), while *Rhodoglobus* and *Nocardioides* were mainly represented in DSI (relative abundance of 16 and 13%, respectively). *Proteobacteria* were mainly represented by *Psychrobacter* in DSII and DSIII (35 and 30%, respectively), while all the other genera showed lowest relative abundance percentages in DSI, ranging from 0.2 to 3%. Overall, *Gillisia* members were predominant among *Bacteroidota* in DS samples (10% for DSI, and 8% for DSII and DSIII), whereas higher abundance of *Antarcticibacterium* was detected only in DSI (relative abundance of 10%). *Firmicutes* members, e.g., *Planococcus* and *Planomicrobium*, were mainly represented in samples DSII and DSIII (relative abundance percentages of 1% for *Planococcus* and 2% for *Planomicrobium*, respectively), *Desulfitibacter*, detected only in sample DSI (relative percentage 1%), and *Desulfosporosinus* almost equally represented in all DS samples.

**Figure 8:**
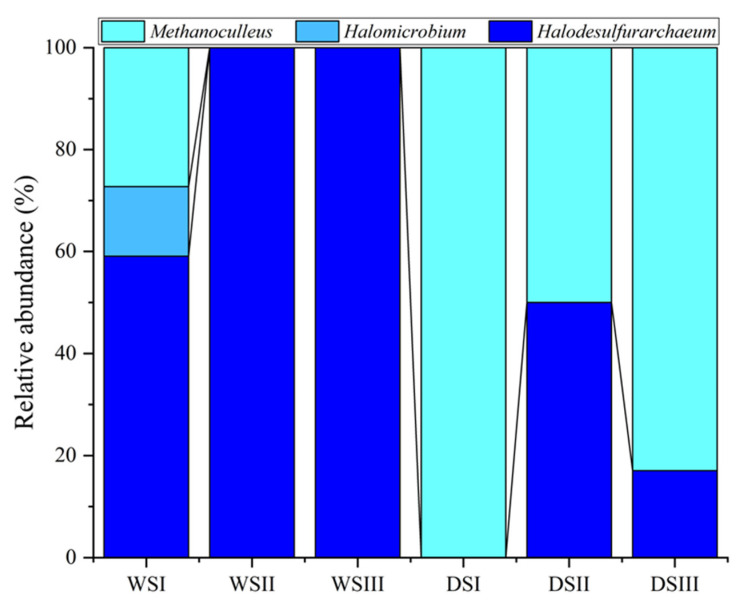
Archaeal taxonomic composition at genus level observed in salt samples. *WS*, white salt; *DS*, dark salt.

The archaeal microbial community in the WS samples contributed to the total microbial community with an abundance percentage lower than 0.1%, ranging from 0.04% to 0.07%. Differently, they accounted for 0.1%, 0.01% and 0.11% in DSI, DSII and DSIII, respectively). Archaea were represented by *Halobacteria* and *Methanomicrobia* members by including at genus level *Methanoculleus*, *Halomicrobium*, and *Halodesulfarchaeum*, differently distributed in the salt samples (Figure 8).

**Figure 9:**
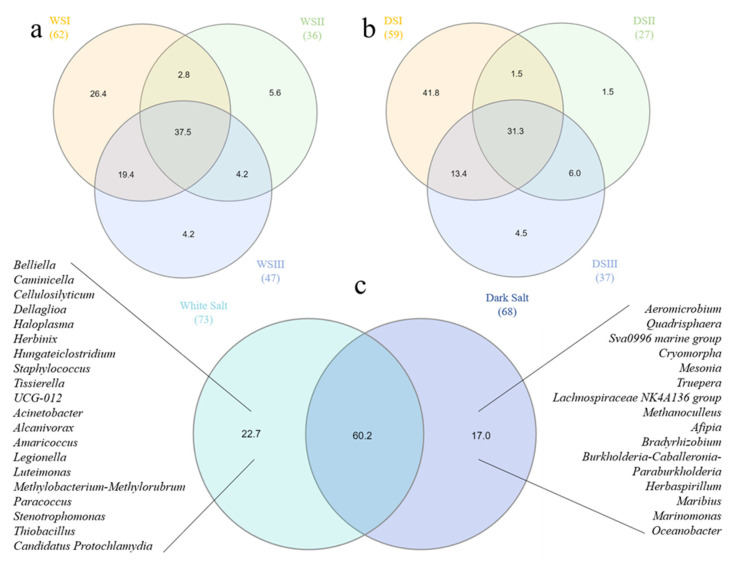
Venn diagram depicting the sharing level of genera retrieved in the total bacterial communities of salt samples. (**a**) *WS* versus *WS*; (**b**) *DS* versus DS; (**c**) *WS* versus *DS*. *WS*, white salt; *DS*, dark salt.

As it is shown in Figure 9a, the ASV-sharing at the genus level between WS samples was 37.5% with the highest value of exclusive genera that was detected for WSI sample (i.e., 26.4%). Similarly, the genus-sharing among DS samples was 31.3%, with DSI presenting a total value of 41.8% of exclusive genera (Figure 9b). The sharing level of genera between WS and DS samples on a whole accounted for 60.2%, with exclusive taxonomic groups accounting for 22.7 and 17% in WS and DS samples, respectively.

### 3.3. Statistical Analyses

**Figure 10:**
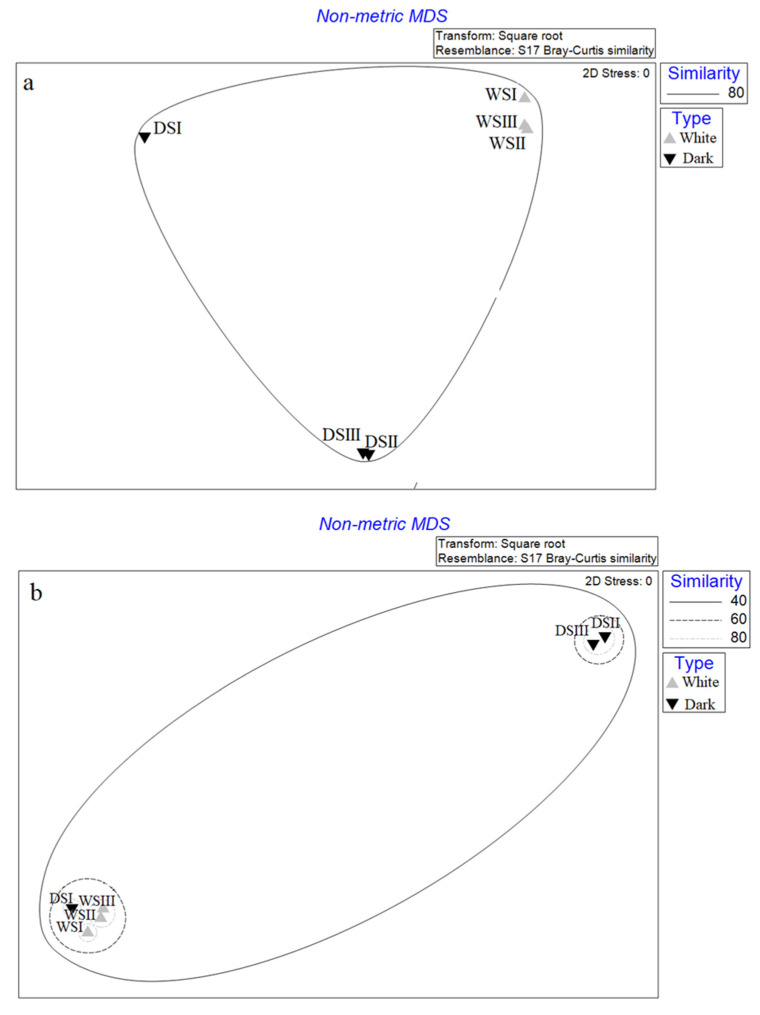
nMDS analysis computed on relative abundance data at phylum (**a**) and genus level (**b**) of bacterial groups. WS, white salt; DS, dark salt.

The nMDS computed with data of relative abundance at the phylum and genus level is shown in Figure 10. In both cases, the WS samples are spatially distributed close together, while among DS samples, DSI appeared isolated by clustering separately from the other two DS samples. At the phylum level, all the samples are grouped in a unique cluster with an overall similarity of 80%, despite the spatial distribution denoting some differences between WS and DS, and in the case of the DSI sample (Figure 10a). At the genus level (Figure 10b), samples DSII and DSIII grouped together in a cluster with 80% of similarity; a second group with a similarity of 60% included a sub-cluster with samples WSII and WSIII (similarity 80%), and samples DSI and WSIII. All salt samples were included in a bigger group with a total similarity of 40%.

## 4. Discussion

Among sodium sulfate minerals (which are strongly dependent on temperature range), the anhydrous phase (i.e., Na_2_SO_4_, thenardite) is generally more common than the two hydrated forms, i.e., Na_2_SO_4_ × 7 H_2_O (sodium sulfate heptahydrate) and Na_2_SO_4_ × 10 H_2_O (mirabilite) [19]. Thenardite and mirabilite extensively occur in nature. According to the classification by Zheng et al. [20], based on the temperature of formation, mirabilite is the most common evaporitic mineral crystallizing under cool temperatures, while thenardite is the typical sodium sulfate produced under warm conditions, commonly found in ancient deposits. Mirabilite is characterized by a low melting point and high solubility, making it a highly reactive mineral. Therefore, only thenardite appears as the prevalent sodium phase in the geological record as ancient deposits [21].

In Antarctica, mirabilite deposits potentially originate from thermal spring waters, oceanic aerosols, entrapped ancient seawater, cryoconcentration of past proglacial lake waters, volcanic emissions, and solubilization of pre-existing salt deposits [7,22]. Both thenardite and mirabilite were previously found in the evaporite deposits at the Lewis Cliff Ice Tongue [7]. The authors reported that temperature-induced changes in the relative humidity probably affected original evaporite minerals, leading to the transition between mirabilite and thenardite. The hydrated state of mirabilite was maintained at a subzero environment in central portions of evaporite mounds, whereas their outer portions dehydrated into powdery thenardite. Mirabilite is also common in the McMurdo Dry Valleys, probably deriving from ancient seawater precipitation as evidenced by isotopic analyses [23], and it was also reported in association with Blood Falls outflow. Lyons et al. [22] hypothesized that mirabilite has been lost from the englacial brine and exists somewhere in the subglacial/englacial system. Boulder Clay Glacier mirabilite and thenardite occurring close to the surface of a debris-cone probably derive by the evaporation of a localized, highly saline water body such as a kettle or ice-marginal lake of the Boulder Clay debris covered glacier as suggested by Chinn [15]. Recently, Cesur et al. [23] demonstrated microbial growth in deliquescent brines in the laboratory and acclaimed the experiment’s relevance for the surface and near-subsoil of cold, arid worlds such as Mars. As conditions become wetter, the hygroscopic minerals from evaporite can deliquefy to produce the first habitable brines witnessing survival after drying and growing in deliquescent brines.

In this study, we first attempted to describe the prokaryotic communities associated with mirabilite/thenardite crystals (fresh and sediment-affected) deriving from the Boulder Clay Glacier supraglacial system. About the origin of the salt cones, they are in a debris covered glacier related to the “Younger Drift” of the surrounding area. Here, they can be generated as ablation/sublimation phenomena associated with the debris-covered dead-ice terrain [11] of the Ross Sea Ice Shelf which is therefore overly mainly salty ice [24,25].

In these harsh environments, the prokaryotic communities are adapted to extreme environmental conditions, deriving from the combination of high irradiation and salinity values, and dryness. Similar conditions might be expected in extraterrestrial habitats, such as the Martian surface. Sulfate, as well as chloride and mineral deposits have been reported as microbial habitats under stressful and limiting environmental conditions [26,27] which can highly limit life. Phylogenetic analysis allowed the debris-cone of four main bacterial lineages, i.e., *Bacteroidota*, *Actinobacteriota*, *Firmicutes*, and *Gammaproteobacteria*. High GC and low-GC bacteria, as well as a number of *Proteobacteria*, often inhabit soil or dust (e.g., *Arthrobacter*, *Methylorubrum*, *Pseudomonas*, *Bradyrhizobium*, *Nocardioides*) thus suggesting that salt samples, being on the surface of a debris-covered glacier, might have received external inputs. Interesting differences were observed between white and dark samples. WS hosted a higher abundance of *Marinobacter* members (ranging from 10.4% to 15.3%, in contrast with DS where they were in the range 1–1.6%) and *Gillisia* (21.4 and 8.7 % in WS and DS, respectively). In WS, the genus *Rhodoglobus* also accounted for double percentages compared to DS. Conversely, *Arthrobacter* and *Psychrobacter* members were more abundant in DS samples, with values ranging from 0.7 to 28.1% and from 0.2% to 35.5%, respectively. The statistical analysis suggested that, even if the communities in WS and DS resembled each other in terms of taxonomic macro-structure, differences occurred and were markedly evident at the genus level. This finding indicates that the two salt strata may receive diversified inputs, e.g., from soil or snow. Moreover, the possible marine origin of the salts is not to be excluded, with an influence that remained more evident in the WS samples.

Differences were also observed within the WS and DS samples. Sample DSI differed from the rest. This was probably due to the total absence of certain taxonomic genera that instead resulted predominantly in DSII and DSIII, namely *Psychrobacter* and *Arthrobacter*. Conversely, genera such as *Antarcticibacterium* and *Rhodoglobus* were better represented in DSI. Due to its markedly different bacterial composition, it is plausible to assume that DSI may have been collected from a sort of transition zone between WS and DS.

Several sequences were related to halotolerant and halophilic genera, reported in a variety of marine environments and saline habitats (e.g., *Halomonas*, *Haloplasma*, *Anaerobacillus*), also in Antarctica. For example, members in the genus *Antarcticibacterium* (family *Flavobacteriaceae*) were isolated from surface sediment collected from the Ross Sea [28]. *Marinobacter*, as well as *Psychrobacter* members were previously detected in cold hypersaline brines and lakes in Antarctica, also in the Northern Victoria Land i.e., [29,30,31,32]. *Gillisia* typically inhabits Antarctic brackish lakes, marine sponges, sea-ice and seawater [33,34,35]. Among less represented genera, *Cyclobacterium* species have been frequently retrieved in seawater, marine sediments, soil from solar salterns [36,37,38,39], and also in cold areas, such as microbial mats from Antarctic lakes [40] and ikaite tufa columns in Greenland [41]. *Thalassospira* members are involved in the carbon cycle in marine environments, and some species have been reported as hydrocarbon degraders that predominate in hydrocarbon-degrading communities from global oceans [42]. Other species, even if less represented in our samples (e.g., *Marinicella*, *Marinomonas*, *Maribius*, *Methyloceanibacter*, *Oceanobacter*, *Hahella* and *Roseimaritima* among Bacteria, and *Halodesulfurarchaeum* and *Halomicrobium* among Haloarchaea), support the origin of the analyzed salt from a saline water body in the BC area. More interestingly, even if it occurred at a lower relative percentage in a unique replicate (namely WSI), *Alcanivorax* is a cosmopolitan obligate marine bacterium, halophilic, that uses a highly restricted spectrum of substrates, predominantly alkanes, as its exclusive source of carbon and energy [43].

As it was expected due to the salt composition, the analyzed salt community included members that are believed to play a major role in the sulfur cycle. Among them, *Sulfurovum*, *Thiobacillus* and *Thiomicrorhabdus* are sulfur-oxidizing bacterial species. In particular, the Antarctic Blood Falls, which are rich in sulfate, host *Thiomicrorhabdus arctica*, highlighting the adaptability of the genus to cold and salt conditions [25]. The detection of known sulfate-reducing bacteria (SRB; i.e., *Desulfosporosinus*, *Desulfitibacter*, *Desulfuribacillus*, *Clostridium*), as well as Archaea (i.e., *Halodesulfurarchaeum*), suggested that biotic sulfate reduction might occur in salt samples. The SRB group is very versatile and includes species that oxidize organic compounds (e.g., acetate) to CO_2_. Moreover, a number of species that are able to autotrophically use CO_2_, H_2_ and sulphates as substrates. *Halodesulfurarchaeum* are obligately anaerobic neutrophilic sulfur-respiring haloarchaea, recently observed in salt lakes, growing by sulfur/thiosulfate-dependent respiration with H_2_ or formate as the electron donors [44,45]. In this regard, the occurrence of strictly anaerobic prokaryotes (e.g., *Halodesulfurarchaeum*, *Petrotoga*, *Thermacetogenium*) suggests anoxic conditions might occur in the original water body alimenting the salt-cone.

With respect to the carbon cycle, hydrogenotrophic methanogens as *Methanoculleus* affiliates, within the family *Methanomicrobiaceae*, live in marine environments and brackish waters [46]. Its co-occurrence in our samples with SRB likely suggest that a syntrophic consortium might occur. The coexistence of SRB and methanogens has been previously observed in other hypersaline environments [47]. Other sequences were related to *Thermacetogenium*, a syntrophically acetate-oxidizing bacterium.

As it was observed in hypersaline brines of a perennially ice-covered lake in Tarn Flat [48], salt samples hosted members related to moderately thermophilic bacteria (e.g., *Thermacetogenium*, *Thermovirga*, *Petrotoga*, *Acetomicrobium*). Even if Antarctica is generally considered as a cold environment, volcanic and geothermal activity occurs in several areas of the continent. No evidence exists about peculiar geothermal activity in the Boulder Clay area, but it not to be excluded that thermophilic microorganisms, or their DNA, may derive from a deep underground circulation of the source water body. Among thermophilic members, *Thermotogota* and *Synergistota* live in hot, anaerobic environments (e.g., hot springs, deep-sea floor, oil reservoirs). Several *Petrotoga* and *Thermovirga* have been reported in association with petroleum wells and reservoirs all around the world e.g., [49,50,51].

## 5. Conclusions

This study has contributed to deepening the current knowledge on the microbial ecology of salt efflorescences from a Boulder Clay Glacier debris-cone in the Northern Victoria Valley (Antarctica). The cryogenic ecosystem studied could be an oasis of life, with differences between the WS and DS layers, where four main bacterial lineages live (*Bacteroidota*, *Actinobacteriota*, *Firmicutes*, *Gammaproteobacteria*). The analyzed salt community in several sequences were related to halotolerant and halophilic genera, hosted members that are believed to play a major role in the sulfur cycle, including members related to moderately thermophilic bacteria. Several species analyzed in the salt are able to autotrophically use CO_2,_ and the occurrence of strictly anaerobic prokaryotes was also detected. Part of the sequences found could represent part of the ancestral microbial communities living in the salt efflorescences or in the briny system of origin. The microorganisms present in the salt efflorescence on earth could be candidates for future implants on distant planets with extreme conditions such as those found in Antarctica.

## Figures and Tables

**Table 1:** Bacterial taxonomic group distribution at genus level (relative abundance ≥ 0.1%). *WS*, white salt; *DS*, dark salt.

Phylum	Genus		WSI	WSII	WSIII	BSI	BSII	BSIII
*Actinobacteriota*	*Aeromicrobium*		0	0	0	0.2	0	0.1
	*Arthrobacter*		1.7	2.1	1.9	0.7	28.1	25.2
	*Cryobacterium*		0.4	0	0	0.4	0	0
	*Cutibacterium*		0.1	0.1	0	0.2	0	0
	*Demequina*		0.2	0.6	0.6	0.8	0.2	0.4
	*Egicoccus*		0	0.3	0.2	0.4	0	0.1
	*Ilumatobacter*		0.2	0	0.2	0	0.1	0.2
	IMCC26207		0.1	0	0.1	0.2	0.1	0
	*Kocuria*		0	0	0	0	0	0
	*Lawsonella*		0	0	0	0	0	0
	*Luteococcus*		0.4	0.2	0.2	0.4	0.2	0.3
	*Mycobacterium*		0.4	0.2	0.2	0.3	0	0.2
	*Nocardioides*		1.8	3	3.4	12.6	2	2.9
	*Quadrisphaera*		0	0	0	0	0.1	0.1
	*Rhodoglobus*		4.2	18.6	14.4	16.4	1.2	3.4
	Sva0996 marine group		0	0	0	0	0	0.2
	*Tessaracoccus*		0.3	0	0.2	0.3	0.1	0.2
*Bacteroidota*	*Antarcticibacterium*		2	9.8	6.3	10.2	2.5	2.3
	*Belliella*		0	3.7	1.5	0	0	0
	*Cryomorpha*		0	0	0	0	0	0.1
	*Cyclobacterium*		0.4	1.1	1	0.8	0.4	0.7
	*Flavobacterium*		0.2	0	0	0.1	0	0
	*Gillisia*		25.4	17.8	20.9	10	8.1	8.1
	*Lentimicrobium*		0.5	0.1	0.2	0.3	0	0.2
	*Mesonia*		0	0	0	0.2	0	0
*Campylobacterota*	*Sulfurovum*		0.3	0	0	0.3	0	0
*Deinococcota*	*Truepera*		0	0	0	0.1	0	0
*Firmicutes*	*Alkalibacterium*		0.2	0	0	0.1	0	0
	*Anaerobacillus*		0.7	0.3	0.4	1	0.2	0.1
	*Bacillus*		0.3	0	0.1	0.2	0	0
	*Caminicella*		0.1	0	0.1	0	0	0
	*Cellulosilyticum*		0.2	0	0.1	0	0	0
	*Clostridium sensu stricto* 13		0.3	0	0.2	0.3	0	0
	*Dellaglioa*		0.2	0	0.1	0	0	0
	*Desulfitibacter*		0.4	0	0	1.1	0	0.3
	*Desulfosporosinus*		3.4	2.6	2.7	1.8	1.2	1.2
	*Desulfuribacillus*		0.1	0	0.2	0.2	0	0
	*Haloplasma*		0.4	0.2	0.2	0	0	0
	*Herbinix*		0	0.1	0	0	0	0
	*Hungateiclostridium*		0.1	0	0	0	0	0
	*Lachnospiraceae* NK4A136 group		0	0	0	0.1	0	0
	*Paenibacillus*		0.1	0	0	0.1	0	0
	*Planococcus*		0.7	0.3	0.3	0	1.4	1
	*Planomicrobium*		0.1	0	0.1	0	2.5	1.9
	*Ruminiclostridium*		0	0	0.3	0.1	0	0
	*Staphylococcus*		0	0	0.1	0	0	0
	*Thermacetogenium*		0.2	0	0	0.4	0	0
	*Tissierella*		0	0	0.1	0	0	0
	UCG-012		0.1	0	0	0	0	0
*Halanaerobiaeota*	*Orenia*		0.9	1.5	1.4	0.3	0.3	0.4
*Halobacterota*	*Methanoculleus*		0	0	0	0.1	0	0
*Myxococcota*	*Enhygromyxa*		0.4	0	0.3	0.3	0	0.1
*Planctomycetota*	*Roseimaritima*		0	0.1	0	0	0.1	0
	*Rubinisphaera*		0.2	0	0	0.4	0	0
*Proteobacteria*	*Acinetobacter*		0.1	0.1	0.1	0	0	0
	*Afipia*		0	0	0	0.2	0	0
	*Alcanivorax*		0.1	0	0	0	0	0
	*Amaricoccus*		0.1	0	0	0	0	0
	*Aureimonas*		0.2	0.2	0.1	0.9	0.2	0.1
	*Blastomonas*		1.3	0.5	0.9	1.4	0.2	0.5
	*Bradyrhizobium*		0	0	0	0.2	0	0
	*Burkholderia-Caballeronia-Paraburkholderia*		0	0	0	0.1	0	0
	*Hahella*		6.7	1	3.3	1.5	0.6	1.6
	*Halomonas*		0.2	0	0	0.2	0	0
	*Herbaspirillum*		0	0	0	0	0	0.1
	*Jannaschia*		0	0.2	0.2	0	0.7	0.3
	*Legionella*		0.8	0.1	0.4	0	0	0
	*Luteimonas*		0.1	0	0	0	0	0
	*Lysobacter*		0	0.1	0	0.3	0	0
	*Maribius*		0	0	0	0.4	0	0.1
	*Marinicella*		0.2	0.3	0.3	0.5	0.2	0.3
	*Marinobacter*		10.4	15.3	14.1	1.6	1	1.4
	*Marinomonas*		0	0	0	0.2	0	0
	*Methylobacterium-Methylorubrum*		0.1	0	0	0	0	0
	*Methyloceanibacter*		0.3	0	0.1	0.2	0	0
	*Oceanobacter*		0	0	0	0.1	0	0
	*Paracoccus*		0	0	0.1	0	0	0
	*Pseudohongiella*		0.1	0	0	0.3	0	0.1
	*Pseudomonas*		5.3	3.5	4	3.2	2.5	3.3
	*Psychrobacter*		0.8	0.5	0.8	0.2	35.5	29.9
	*Salinarimonas*		0.5	0.2	0.3	0.8	0.3	0.3
	*Stenotrophomonas*		0.1	0	0	0	0	0
	*Thalassospira*		0.6	0.1	0.2	0.7	0.2	0.3
	*Thiobacillus*		0.2	0.1	0	0	0	0
	*Thiomicrorhabdus*		0.5	0	0.1	0.5	0	0
*Synergistota*	*Acetomicrobium*		0.4	0.1	0.2	1.6	0	0.3
	*Thermovirga*		0.4	0	0	0.5	0	0
*Thermotogota*	*Petrotoga*		0.6	0	0.2	1.9	0	0
*Verrucomicrobiota*	*Cand. Protochlamydia*		0.1	0	0	0	0	0
	*Luteolibacter*		0	0.1	0	0.2	0	0
Not Assigned			17.2	10	12	17.8	6	7.2
*n* = 0	0.1 < *n* < 1	1.1 < *n* < 5	5.1 < *n* < 10		10.1 < *n* < 20		*n* > 20	

## Data Availability

All sequences were submitted to the National Center for Biotechnology Information (NCBI) under the accession numbers SAMN29936604 (WSI), SAMN29936605 (WSII), SAMN29936606 (WSIII), SAMN29936607 (DSI), SAMN29936608 (DSII), SAMN29936609 (DSIII).

## Supplementary Materials

The following supporting information can be downloaded at https://www.mdpi.com/article/10.3390/microorganisms10091753/s1, Table S1: Total number of sequence reads, good quality reads, observed numbers of ASVs, Shannon diversity, Evenness and Chao 1 indices per sample of the 16S rRNA gene data sets. WS, white salt; DS, dark salt. Figure S1: Rarefaction curve calculated and plotted by the R package “iNEXT” and the package ggplot2. The diversity estimates were calculated for the sample size determined by the endpoint and the specified predefined nodes, furthermore, the endpoint was set as double the reference size, and the number of replicates was set to 50. WS, white salt; DS, dark salt.
