# Supplementary material for: Antarctic Salt-Cones: An Oasis of Microbial Life? The Example of Boulder Clay Glacier (Northern Victoria Land)
Source: Microorganisms. 2022 Aug 30;10(9):1753. doi: 10.3390/microorganisms10091753 (PMC9504174; doi:10.3390/microorganisms10091753)
Supplement: Supplementary file 1 [file microorganisms-10-01753-s001.zip › microorganisms-1864057-supplementary.pdf]

microorganisms

Supplementary Materials

Antarctic salt-cone: an oasis of microbial life? The example of Boulder Clay Glacier (Northern Victoria Land)

Maurizio Azzaro [1], Maria Papale [1], Carmen Rizzo [1,2], Emanuele Forte [3], Davide Lenaz [3], Mauro Guglielmin [1,4] and Angelina Lo Giudice [1,*]

[1] Institute of Polar Sciences, National Research Council (CNR-ISP), Messina (Italy); maurizio.azzaro@cnr.it (M.A.); maria.papale@isp.cnr.it (M.P.); angelina.logiudice@cnr.it (A.L.G.)
[2] Stazione Zoologica "Anton Dohrn", Marine Biotechnology Department, Sicily Marine Centre, Villa Pace, Messina, Italy; carmen.rizzo@szn.it (C.R.)
[3] Department of Mathematics and Geosciences, Trieste University, Italy; eforte@units.it (E.F.); lenaz@units.it (D.L.)
[4] Department of Theoretical and Applied Sciences, Insubria University, Varese, Italy; mauro.guglielmin@uninsubria.it (M.G.)

* Correspondence: angelina.logiudice@cnr.it; Tel.: 00396015414

Supplementary Table S1. Total number of sequence reads, good quality reads, observed numbers of ASVs, Shannon diversity, Evenness and Chao 1 indices *per* sample of the 16S rRNA gene data sets. WS, white salt; DS, dark salt.

Sequence data	*Dark salt*			*White salt*		
	DSI	DSII	DSIII	WSI	WSII	WSIII
Total Reads	73525	37748	83325	89727	83953	33844
GC (%)	53	54	52	54	53	53
Good Quality Reads (%)	84.6	84.6	87.1	86.1	85.3	84.9
ASVs	582	433	521	701	604	499
Shannon	4.68	3.31	3.67	4.72	4.08	4.37
Inv simpson	0.97	0.89	0.91	0.97	0.96	0.97
Evenness	0.18	0.06	0.07	0.16	0.09	0.15

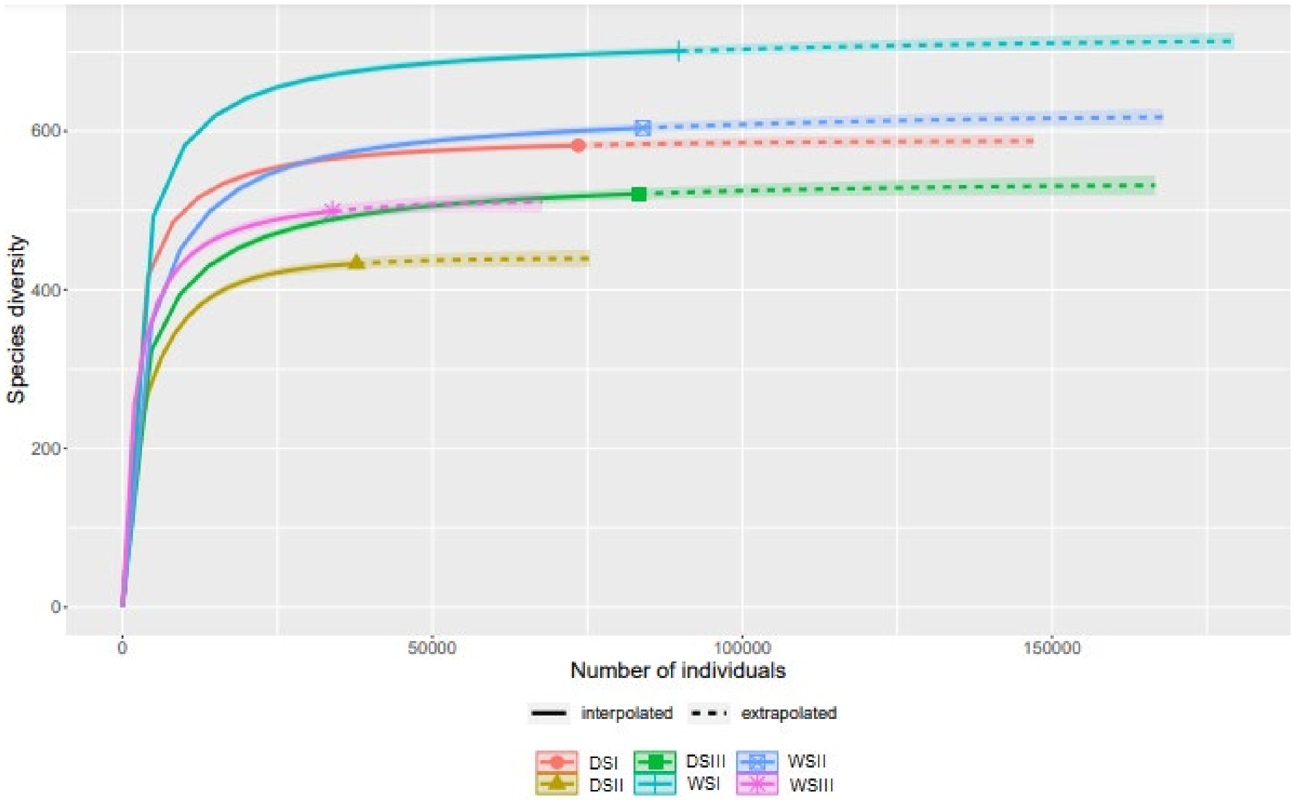

Supplementary Figure S1. Rarefaction curve calculated and plotted by the R package "iNEXT" and the package ggplot2. The diversity estimates were calculated for the sample size determined by the endpoint and the specified predefined nodes, furthermore, the endpoint was set as double the reference size, and the number of replicates was set to 50. *WS*, white salt; *DS*, dark salt.
